# In This Issue

**DOI:** 10.1002/cfg.440

**Published:** 2004


**Comparative and Functional Genomics**
*Comp Funct Genom* 2004; **5**: 457–458.
Published online in Wiley InterScience (www.interscience.wiley.com). **DOI:** 10.1002/cfg.440

### In this issue

### Identifying pathways involved in cardiac hypertrophy

In their paper, **Strøm *et al.*** describe the identification of pathways with roles in cardiac hypertrophy using microarrays to profile a range of models of this disease. Their experiments have uncovered a core set of hypertrophy-specific genes which have consistent differential expression in all of the models of this disease.

### Navigating public microarray databases

**Christopher Penkett and Jürg Bähler** review the wide range of public databases that are currently available for storage and analysis of microarray data. They discuss the role played by smaller, specialised databases and the case for comprehensive public repositories.

### Meeting Review: Predicting the structure of biological molecules

**Damian Counsell** reviews the presentations made at a structural bioinformatics workshop held in Cambridge, UK in April this year. The workshop brought together the cream of UK structural bioinformatics, providing a fresh and diverse view of progress in biomolecular structure prediction and analysis, and protein evolution studies.

### Meeting Review: Genomes 2004

The Genomes 2004 conference gathered researchers working on a wide range of microbial genomes, with the aim of sharing their latest observations and insights. **Jo Wixon** reports on three of the sessions 'Genome analysis and comparative genomics', 'Computational genomics' and 'Functional genomics'.

### Special section of papers from the Bio-Ontologies special interest group (SIG) meeting from ISMB 2004

**Phillip Lord and Robert Stevens** open this special section with a report from the Bio-Ontologies meeting and an analysis of the current state of Bio-Ontology studies.

Medical practitioners and informaticians are primarily interested in structures, functions and processes at coarser levels of granularity than those which interest theoretical biologists and bioinformaticians. **Anand Kumar *et al.*** discuss how a theory of granular levels could play an important role in bridging the gap between these two groups of disciplines.

The ontologies comprising the Open Biological Ontologies (OBO) project are formalizations of various domains of biological knowledge. **Chris Mungall's** paper describes an effort to elucidate the language of definitions encoded in the terms in these ontologies (called Obol), with the aim of enabling computers to reason over the resulting definitions.

The annotation of much functional genomics data and of large-scale *in situ* gene expression or phenotype screen data would benefit from a minimum set of standard anatomical terms. **Helen Parkinson *et al.*** describe the SOFG Anatomy Entry List, a simple, accessible, controlled vocabulary of gross anatomical terms, produced by a Standards and Ontologies for Functional Genomics (SOFG) group in collaboration with several major anatomical ontologies for higher vertebrates.

**Satoko Yamamoto and colleagues** present the Molecule Role Ontology, an ontology designed to annotate molecules in the scientific literature on signal transduction pathways. This ontology will assist in the development of a reliable pathway database from information gathered from the scientific literature.

Automated genome comparison is a complex task, requiring formal descriptions and semantics of domain terms to be defined in order to enable logical reasoning. **Keith Flanagan *et al.*** describe

Copyright © 2004 John Wiley & Sons, Ltd.

an ontology for describing genomes, genome comparisons, their evolution and biological function. This ontology will facilitate consistent annotation of genomes through computational methods, supporting the development of genome comparison algorithms.

**Georgios Gkoutos and colleagues** outline a schema that can be used to describe phenotypes by combining orthologous axiomatic ontologies. They have produced tools for storing, browsing and searching such complex ontologies and have tested their approach on a large set of mutant mouse phenotypes detected using the SHIRPA protocol, a well-defined collection of behavioural assays.

### Conference Calendar

A listing of genomics related conferences planned for February to April 2005.

Copyright © 2004 John Wiley & Sons, Ltd.

